# Cultural selection drives the evolution of human communication systems

**DOI:** 10.1098/rspb.2014.0488

**Published:** 2014-08-07

**Authors:** Monica Tamariz, T. Mark Ellison, Dale J. Barr, Nicolas Fay

**Affiliations:** 1 School of Philosophy, Psychology and Language Sciences, University of Edinburgh, Edinburgh EH8 9LL, UK; 2 School of Psychology, University of Western Australia, Crawley, Western Australia 6009, Australia; 3 Institute of Neuroscience and Psychology, University of Glasgow, Glasgow G12 8QB, UK

**Keywords:** cultural evolution, language evolution, drift, coordination bias, content bias, selection

## Abstract

Human communication systems evolve culturally, but the evolutionary mechanisms that drive this evolution are not well understood. Against a baseline that communication variants spread in a population following neutral evolutionary dynamics (also known as drift models), we tested the role of two cultural selection models: coordination- and content-biased. We constructed a parametrized mixed probabilistic model of the spread of communicative variants in four 8-person laboratory micro-societies engaged in a simple communication game. We found that selectionist models, working in combination, explain the majority of the empirical data. The best-fitting parameter setting includes an egocentric bias and a content bias, suggesting that participants retained their own previously used communicative variants unless they encountered a superior (content-biased) variant, in which case it was adopted. This novel pattern of results suggests that (i) a theory of the cultural evolution of human communication systems must integrate selectionist models and (ii) human communication systems are functionally adaptive complex systems.

## 1. Introduction

Human communication systems, such as language, evolve culturally [1,2]; the diverse range of words and grammatical forms used in today's language families can be traced back to a common ancestor [3–7]. The precise mechanism behind the spread of communicative variants, however, is not clear. Neutral evolution (also known as ‘drift’) models have been used to explain the evolution of human communication systems [8–10], and cultural evolution more generally [11,12]. Under this account, cultural change is unbiased: for instance, vocabulary, baby names [11] and pottery designs [12] have been found to spread through random copying. This is a *neutral* account because all variants encountered are considered equal candidates for copying. This paper shows that drift alone is insufficient to explain the evolution of human communication systems. We demonstrate that selectionist cultural evolutionary pressures are necessary to fully explain the rapid propagation of communication variants in a population of interacting human agents.

In any finite evolving population, the frequencies of different variants are affected by drift, but not by selectionist forces; for this reason, drift can be taken as the null hypothesis against which selection can be tested [13–15]. While drift is the null hypothesis for several models of cultural evolution [8–10], it does not always adequately explain empirical results [10,16]. In alternative cultural-selectionist models variant adoption is biased. Theoretical models of human communication argue that during conversation interlocutors are biased to adopt the same labels and other aspects of linguistic representation (including prosody and syntax) [17]. This alignment mechanism has been extended by computer simulation to account for the emergence of linguistic conventions: when agents are biased to match the linguistic behaviour of their interlocutor, a single variant can propagate across a population of interacting computer agents [18,19]. This behaviour-matching account operates at the level of the individual. We call it the *coordination-biased model*. Under a different selection account, called content-biased selection [20,21], functional selection [10] or replicator selection [16], variant adoption depends upon the intrinsic value of the particular variant. For example, variants that are easier to learn or use have an increased likelihood of being adopted, and therefore propagate in populations faster than a neutral drift model would predict. This second alternative account operates at the level of the cultural variant. Following Boyd & Richerson [20], we call it the *content-biased model*. For a discussion of the other types of cultural bias that can affect social learning, see [20,22]. Against a baseline drift model, this paper tests the coordination- and content-biased selection models’ ability to explain the spread of communication variants in an experimental micro-society. It examines for the first time the explanatory power of each evolutionary account and the interplay between them before concluding that a theory of the cultural evolution of human communication systems must integrate the two selectionist models.

Laboratory experiments are being increasingly used to study the mechanisms that underpin cultural evolution (for reviews, see [23,24]). By virtue of their ability to control and manipulate variables of interest, experiments allow researchers to test specific hypotheses about the social learning mechanisms critical to cultural change. Artificial language learning studies have been used to study the evolution of language-like structure [25–27], and experimental-semiotic studies have been used to study the evolution of sign systems [28–32]. This paper models the results of an experimental-semiotic study, where human participants communicate a set of fixed concepts by drawing on a shared digital whiteboard [29]. In this paradigm, participants are not allowed to use conventional language (spoken or written), forcing them to create a new graphical communication system from scratch (for a review, see [33]). Participants were organized into four 8-person micro-societies and communicated a list of recurring concepts (e.g. *art gallery, drama, theatre*; see the electronic supplementary material, S1 for a full listing) to their partner (i.e. all communication took place in pairs). After several games, they switched partners and continued in this way until they had interacted with each of the other members of their group.

**Figure 1.**
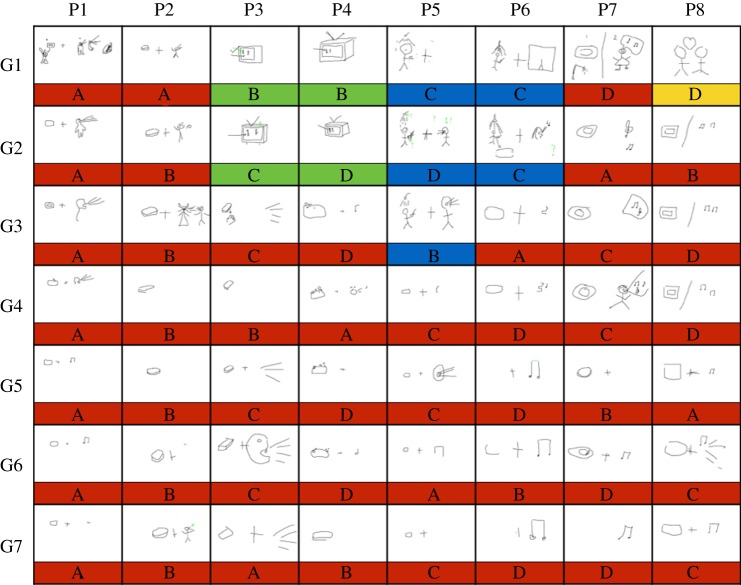
Cultural evolution of the signs used to represent *soap opera* in an 8-person micro-society (from [29]). Columns correspond to participants (P1–P8) and rows to generations (G1–G7). Capital letters (AA, BB, CC, DD) indicate the different participant pairings in a given generation, and colours indicate the different variant types. When participants played with their first partner (generation G1), they used a variety of different signs: a bar of soap and a musical note (red variant), a television (green), a shower (blue) and a love heart (yellow). At generation G1, participants tended to adopt the sign their partner produced. As they interacted with the other members of their micro-society, the soap and musical note (red variant) propagates until everyone is using a refined version of this sign (either soap, a musical note or both) by generation G4. Note that participants retain their initial variant until they encounter the soap and musical note variant (red), after which they only use this variant. This suggests a strong content bias for the soap and musical note variant (red) such that it was more likely to be adopted by participants compared with its competitors in this particular micro-society. In each micro-society, participants communicate 16 concepts, giving 64 distinct data structures like the above (a total of 3584 signs).

Within each micro-society, sign variation was lost as members of the group aligned on a uniform inventory of single sign-to-meaning mappings. A representative example of the spread of a cultural variant for the concept *soap opera* within a micro-society is given in figure 1. Across micro-societies sign variation increased: different micro-societies aligned on different inventories of sign-to-meaning mappings. To be clear, there was no common pattern in the communication systems that evolved across the different isolated micro-societies; different micro-societies developed different ‘dialects’. Analogous to the great variety of human languages [34], sign diversity was a defining outcome of communication in the separate populations.

What cultural evolutionary dynamics best explain the change in frequencies of the communication variants within each experimental micro-society? To answer this question, we constructed a model that mirrored the structure and pattern of interactions of the experimental micro-societies collected by Fay *et al*. [29]. This model included parameters for coordination bias, content bias, memory size and mutation; the drift baseline condition is modelled by setting the coordination- and content-bias parameters to zero. The fit of possible parameter combinations was then assessed against the empirical data. Simulating the behaviour of corpus data collected under controlled laboratory conditions minimizes the effect of extraneous variables and increases our confidence in the explanatory power of the model.

## 2. Material and methods

### (a) Data

The data to be evaluated, collected by Fay *et al*. [29], are structured by micro-society (the four distinct 8-person groups) and by concept (the 16 concepts in electronic supplementary material, S1), yielding a total of 64 data structures like the one illustrated in figure 1. Each data structure includes 56 representations of the concept: one drawing per participant in each of the seven generations. The 56 representations in each data structure were classified into distinct variants (denoted by colour), reflecting common features and structure. The modelled data are available at http://comlab.me/ComLab/Selection.html.

**Figure 2.**
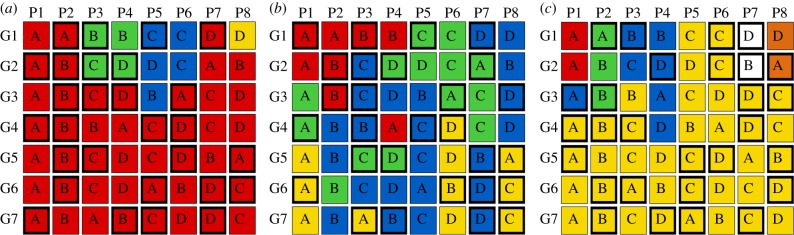
Three data structures that reflect the changing frequencies of the variants used to communicate *soap opera* (panel (*a*), from figure 1), *microwave* (panel (*b*); see the electronic supplementary material, S2) and *Brad Pitt* (panel (*c*); see the electronic supplementary material, S3). Columns correspond to participants (P1–P8) and rows to generations (G1–G7). Capital letters (A with A, and so on) indicate the different participant pairings in a given generation, and colours indicate the different variant types. Boxes with a solid border indicate the participant that drew second in their pair.

The first step of coding each data structure established the initial state of the communication system, labelling the distinct variant types at generation 1. In the case of figure 1, four variant types were identified (red, green, blue and yellow). Because a variety of distinct signs were used to communicate each meaning, and because different micro-societies used different signs to communicate the same meaning, a unique coding scheme was developed for each data structure. The substantial sign variation made coding the different variants at generation 1 straightforward. The second step tracked the spread of the variant types across the subsequent six generations taking into account similarity and descent (whether the producer of a variant had seen that variant before and therefore could be reproducing it rather than independently inventing it). The 64 data structures were coded in this way by two coders (T.M.E. and N.F.), and a third coder (M.T.) resolved any coding conflicts (nine coding conflicts arose: 14.06% of the data structures, and 0.0025% of the variants). Three illustrative coded data structures are shown in figure 2.

Many data structures started off with a large number of variant types and lost diversity over the generations, but others had very little diversity from the start, and in yet others, mutation introduced variability at later generations. Table 1 quantifies this heterogeneity by showing the change in the number of variant types over generations 1–7.

**Table 1.** Descriptive information about the number of variant types found at each generation in the 64 data structures.

	G1	G2	G3	G4	G5	G6	G7
mean	4.19	3.23	2.55	2.25	1.92	1.81	1.64
s.d.	1.39	1.22	1.08	1.07	0.93	0.87	0.76
max.	7	6	5	5	4	4	3
min.	1	1	1	1	1	1	1

### (b) Model

We constructed a parametrized model of participant variant choice. The model takes as input the history of the representational variants the participant had used or seen a partner use and returns a distribution over how they might next represent that concept. The model takes four parameters as described below.

*Memory size* (*m*). Simulations of cultural evolution have shown that a smaller memory for experienced past variants promotes more rapid population-level convergence on a single communication variant [19]. So the model includes a parameter indicating the maximum amount of history that can influence the variant choice. Each variant found in the history is marked as either produced by the participant (E for *ego*), or by one of their partners (A for *allo*). A memory size of *m* means that the model remembers at most the last *m/*2 E-entries *h_|E,m_* and the last *m/*2 A-entries *h_|A,m_* from any history *h*. The relative frequencies of variants in *h_|E,m_* define the egocentric initial distribution *f*(*h_|E,m_*) and in *h_|A,m_* the allocentric distribution *f*(*h_|A,m_*). Here, *f* maps a list onto the relative frequencies of items in that list. Memory sizes of 2, 4, 6 and 8 were examined.

*Coordination bias* (*c*) fixes the likelihood of being copied ascribed to variants produced by others and witnessed by the participant, and the variants produced by the participant themself. It takes values ranging from −1 (fully egocentric: preferring self-produced variants over other-produced variants) to +1 (fully allocentric: preferring other-produced variants over self-produced variants). Zero coordination bias treats variants in *h_|E,m_* and in *h_|A,m_* as equally worthy of reproduction, i.e. unbiased. For brevity, we sometimes use an affine transformation *γ* = (*c* + 1)/2 of coordination bias as an equivalent parameter. Coordination bias values from −1 to 1 in steps of 0.2 were examined.

*Content bias* (*τ*, *b*) comprises two parameters: the *target *τ* of the content bias, identifying which variant is intrinsically preferred over the others, and the *level b*, which determines how much the target variant is preferred over its peers. If the target variant is not in memory, content bias is ineffective—you need to be familiar with a possible representation before you can prefer it. For notational convenience, we will use *β* = *β*(*τ*, h|_m_*) as equal to *b* whenever *τ* is found in 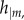 but 0 otherwise. This value is one of the coefficients to the singleton distribution 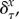 which is 1 if 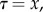 and zero otherwise. The null hypothesis is that the content bias has level 0, i.e. there is no variant preference. When there is non-zero content bias and the biased variant is available in memory, the existing distribution is scaled by (1 − *β*) and then *β* is added to the probability of the target element *τ*. Content bias takes values from 0 to 1 in steps of 0.1.

A drift model has a coordination bias of 0 and a content bias of 0.

*Mutation rate* (*μ*). On rare occasions, participants generate novel variants not seen in their history. To capture this possibility, the combined distribution is linearly combined with a flat distribution *φ*(*x*) weighted by the mutation rate. If the mutation rate is 2%, then 98% of variant choices will reflect the combined distribution, while 2% will be a random choice among all possible variants. The mutation rate is a constant parameter, fixed across all communities, participants and concepts. The rate of new, original variants in the data in [29] was found to consistently fit a mutation rate of 2%, so the parameter was fixed at this value.

Together the parameters define the probability distribution shown in equation (2.1), varying over potential representational variants *x*, for a given history *h*. The overbar is used for probabilistic complement, i.e. 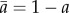.2.1
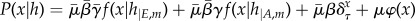


Table 2 summarizes the different levels of each parameter examined. Content bias and coordination bias cover the entire range of possible values, while (as noted above) mutation is fixed at a single value. While, theoretically, memory size could take on larger values, simulations showed that no additional explanatory value was added by increasing memory size beyond 8.

**Table 2.** Levels of each parameter examined.

type	variable	no. levels	levels
explanatory	content bias	11	*b* = 0.0 to 1.0 in steps of 0.1 *τ* = 1, 2, 3, 4, 5, 6, 7, 8
explanatory	coordination bias	11	*c* = −1.0 to 1.0 in steps of 0.2
control	memory	4	*m* = 2, 4, 6, 8
control	mutation	1	*μ* = 0.02

The conditional probability of an entire data structure for a given parameter setting is the product of the probabilities of each choice made (ignoring the first generation, where variant choice cannot be predicted). These probabilities depend on the history of the participant making the choice, and on the model parameters. For each data structure, an exhaustive search was performed over the values shown in table 2 to find the maximum likelihood. The parameter combinations with maximum likelihood were identified.

An example of how a model assigns a probability to a single variant choice is given in the electronic supplementary material, S4. Electronic supplementary material, S5 extends this to the calculation of the likelihood of an entire data structure.

## 3. Results

The values given in table 2 define 484 possible points in the parameter space. The likelihood of each of the 64 data structures was evaluated at each point, and the best parameter setting was retained. The strength of evidence for a bias in particular data structures was evaluated using a best-account Bayes' factor: the maximum likelihood of any model with the bias divided by the maximum likelihood of any model without the bias. This approach is formally equivalent to Kass & Raftery's [35] use of Bayes' factor, although the thresholds for different strengths of support differ slightly. Although Kass & Raftery [35] count *strong support* from a Bayes factor of 20, our threshold for *significant evidence* (in keeping with the standard *p* < 0.05 significance criterion) is 19.

**Figure 3.**
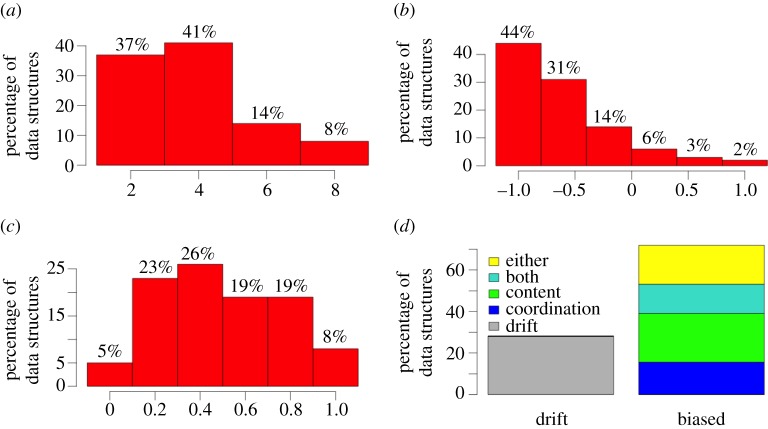
Histograms showing the frequency of different levels of (*a*) memory size, (*b*) coordination bias and (*c*) content bias in the best-fit models for the 64 data structures. (*d*) Shows the number of data structures that are explained by the biases. These break down into four types: 23% show significant evidence for content bias but not coordination bias (green), 16% show coordination (in this case an egocentric bias) bias without content bias (blue), 14% have significant evidence for both (turquoise) and the remaining 19% show significant evidence for the presence of some bias, but there is insufficient evidence to pinpoint which biases are active (yellow).

Lower memory size (2 or 4) was associated with better model fit. Contrary to a coordination bias, an egocentric bias (−1.0 to −0.5), where agents tend to re-use variants they have used previously, was associated with better model fit. Most data structures are best accounted for with some content bias (95% of data structures). Although 28% of the data structures are not distinguishable from a baseline drift account, 72% of the data structures require a biased account (coordination and content; figure 3). Although the median Bayes' factor for coordination bias alone and content bias alone is below the significant evidence criterion of 19 (6.03 and 14.11 respectively), together they returned a median value of 71.52. This indicates a critical interplay between the biases: people tend to re-use variants they have used in the past unless the newly encountered variant is superior, in which case it is adopted (because the content bias typically overwhelms the egocentric bias).

## 4. Discussion

Using simulations to model the spread of communication variants in several experimental micro-societies, we extend neutral models to show that selection models (coordination- and content-biased) play a crucial role in the cultural evolution of human communication systems. Our key finding concerns the symbiotic interplay between the two biases evaluated. The conservative egocentric bias preserves sign variation by inhibiting the adoption of variants produced by others (a similar pattern is observed in natural language, [36]). This bias on its own acts against the convergence of the population onto a shared inventory of signs. The content bias is opportunistic: it encourages variant adoption on the basis of the intrinsic qualities of the encountered variant; if the newly encountered variant is superior to previously used variants it is adopted. The biases in combination maximize the chance that a population will converge on the best variant available to them. To this end, the egocentric bias acts to preserve sign variation, giving the overriding content bias a larger, more competitive inventory of signs to select from. This finding supports a novel mechanism through which populations converge on a shared inventory of sign-to-meaning mappings.

Our findings agree with theoretical and experimental studies of human communication showing that people tend to align their linguistic representations [17,37,38]. However, it questions the mechanism through which alignment is achieved. Rather than consider alignment to be a result of a low-level coordination bias, our findings suggest that alignment can also be driven by higher-level selection in tandem with a resistance to alignment in the form of an egocentric bias. This interplay between content and egocentric biases may be especially pertinent in the earliest stages of language evolution when interlocutors do not already share an inventory of signs. Furthermore, our empirically grounded simulations suggest an alternative to agent-based simulations that show population-level convergence occurs through reinforcement learning [18] or a coordination bias [19] (for a review, see [39]).

If a content bias affects the spread of communication variants in a population, then this would be reflected by the improved functional adaptation of the selected variants. Two experimental studies examined the intergenerational transmission of the communication systems modelled in this paper (i.e. the same corpus of variants collected by Fay *et al*. [29]). One study [40] examined sign adaptation for comprehension. Using a speeded recognition task, it showed that the selected signs (generation G7) were decoded faster and more accurately by naive learners than the initial signs (generation G1). Another study [41] examined sign adaptation for production. It showed that the selected signs (generation G7) conferred specific production advantages for naive learners: they were quicker to recall, were more rapidly executed and were reproduced with higher fidelity than the initial signs (generation G1). This type of functional adaptation is precisely what would be predicted if a content bias were operating on the communication variants.

A key innovation of this study is modelling the cultural spread of communication variants within a small-scale and tightly constrained experimental environment. This complements the modelling of cultural phenomena within large-scale naturalistic datasets [11,16]. A concern with the latter approach is its reliance on data derived from an unconstrained environment, where multiple distinct biases (e.g. content, model and frequency bias [20]) may obscure one another, or unanticipated patterns in the data may be overlooked in the absence of a clear explanation (e.g. a spike in the frequency of a particular variant such as a baby name or dog breed, [42,43]). Although ecological validity may be compromised, modelling the change in the frequency of cultural variants produced in an experimental setting permits a higher resolution test of the effect of specific cultural biases within a smaller, but less noisy, dataset.

There are of course other types of bias that may affect the spread of communication variants in a population. For instance, people selectively copy the linguistic behaviour of those who display traits that are perceived as desirable [44]. This type of ‘model bias’ [20] could not influence the data collected by Fay *et al*. [29], because participants communicated anonymously across a computer network. A ‘conformity bias’ reflects the tendency for people to match their behaviour to the group norm [45,46]. This study helps explain why particular variants propagate in a population, at which point a conformity bias can also apply [47]. While the visual modality offers benefits for communication over the auditory modality [31,48], we do not expect differences between modalities to affect the results presented in this paper [49].

In conclusion, some of the modelled data cannot be distinguished from neutral drift. Crucially, the majority of our results indicate an important interplay between content and egocentric biases in explaining the evolution of human communication systems. Accepting that selection pressures drive the spread of communication variants supports the view that human communication systems are functionally adaptive complex systems [50].

## Supplementary Material

Tamariz et al SM1

## Supplementary Material

Tamariz et al SM2

## Supplementary Material

Tamariz et al SM3

## Supplementary Material

Tamariz et al SM4

## Supplementary Material

Tamariz et al SM5
