# Supplementary material for: Cultural selection drives the evolution of human communication systems
Source: Proc Biol Sci. 2014 Aug 7;281(1788):20140488. doi: 10.1098/rspb.2014.0488 (PMC4083785; doi:10.1098/rspb.2014.0488)
Supplement: Tamariz et al SM1 [file rspb20140488supp1.pdf]

SUPPLEMENTARY MATERIALS 1

The set of 16 target concepts that directors communicated to matchers in [26] (the 4 distracter concepts given in italic).

Places	People	Entertainment	Objects	Abstract
Art Gallery	Arnold Schwarzenegger	Cartoon	Computer Monitor	Homesick
Parliament	Brad Pitt	Drama	Microwave	Loud
Museum	*Hugh Grant*	*Sci-Fi*	*Refrigerator*	Poverty
Theatre	Russell Crowe	Soap Opera	Television	*Sadness*
