# Supplementary material for: Cultural selection drives the evolution of human communication systems
Source: Proc Biol Sci. 2014 Aug 7;281(1788):20140488. doi: 10.1098/rspb.2014.0488 (PMC4083785; doi:10.1098/rspb.2014.0488)
Supplement: Tamariz et al SM2 [file rspb20140488supp2.pdf]

SUPPLEMENTARY MATERIALS 2

Cultural evolution of the signs used to represent *Microwave* in an 8-Person micro-society (from [29]). Columns correspond to Participants (P1 to P8) and rows to Generations (G1 to G7). Capital letters (A with A, and so on) indicate the four different participant pairings in a given generation and colours indicate the different variant types. At Generation 1 three similar but discriminable variants were used to communicate *Microwave*: a three-dimensional drawing of a microwave (red variant), a 2-dimensional drawing of a microwave with food (green variant), and a two-dimensional drawing of a microwave (blue variant). By Generation 4 the red variant has been extinguished, the blue variant has become more frequent than the green variant and a mutation is introduced by Person 6, giving a yellow variant (radiation waves). By Generation 7 the green variant has been extinguished, and the blue and yellow variant are equally frequent. This data structure suggests a weak content bias in favor of the yellow variant.

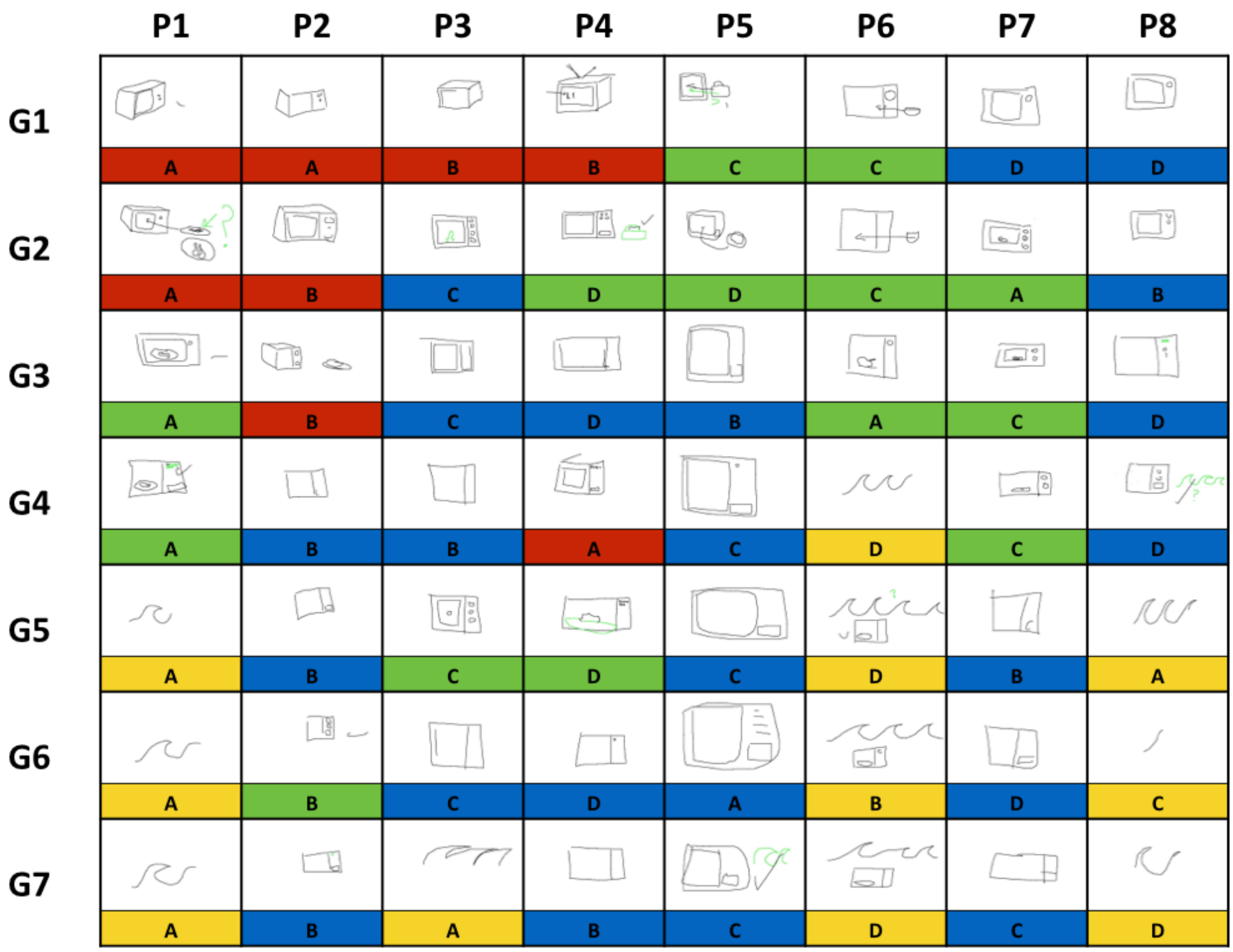
P1 P2 P3 P4 P5 P6 P7 P8
G1 A A B B C C D D
G2 A B C D D C A B
G3 A B C D B A C D
G4 A B B A C D C D
G5 A B C D C D B A
G6 A B C D A B D C
G7 A B A B C D C D
