# Supplementary material for: Cultural selection drives the evolution of human communication systems
Source: Proc Biol Sci. 2014 Aug 7;281(1788):20140488. doi: 10.1098/rspb.2014.0488 (PMC4083785; doi:10.1098/rspb.2014.0488)
Supplement: Tamariz et al SM3 [file rspb20140488supp3.pdf]

SUPPLEMENTARY MATERIALS 3

Cultural evolution of the signs used to represent *Brad Pitt* in an 8-Person micro-society (from [29]). Columns correspond to Participants (P1 to P8) and rows to Generations (G1 to G7). Capital letters (A with A, and so on) indicate the four different participant pairings in a given generation and colours indicate the different variant types. At Generation 1 six different variants were used to communicate *Brad Pitt* across the participants. These included an American flag (red variant), a man and woman (blue variant) a globe (orange variant) and one pair used the rebus principle to represent part of the test concept (a hole in ground to convey a 'pit'; yellow variant). As participants interacted with the other members of their micro-society, the 'pit' sign (yellow variant) propagates until everyone is using a version of this sign by Generation G5. With the exception of Person 4, each participant immediately adopted the 'pit' sign (yellow variant) after they encountered it. This suggests a strong Content bias for the 'pit' sign (yellow variant).

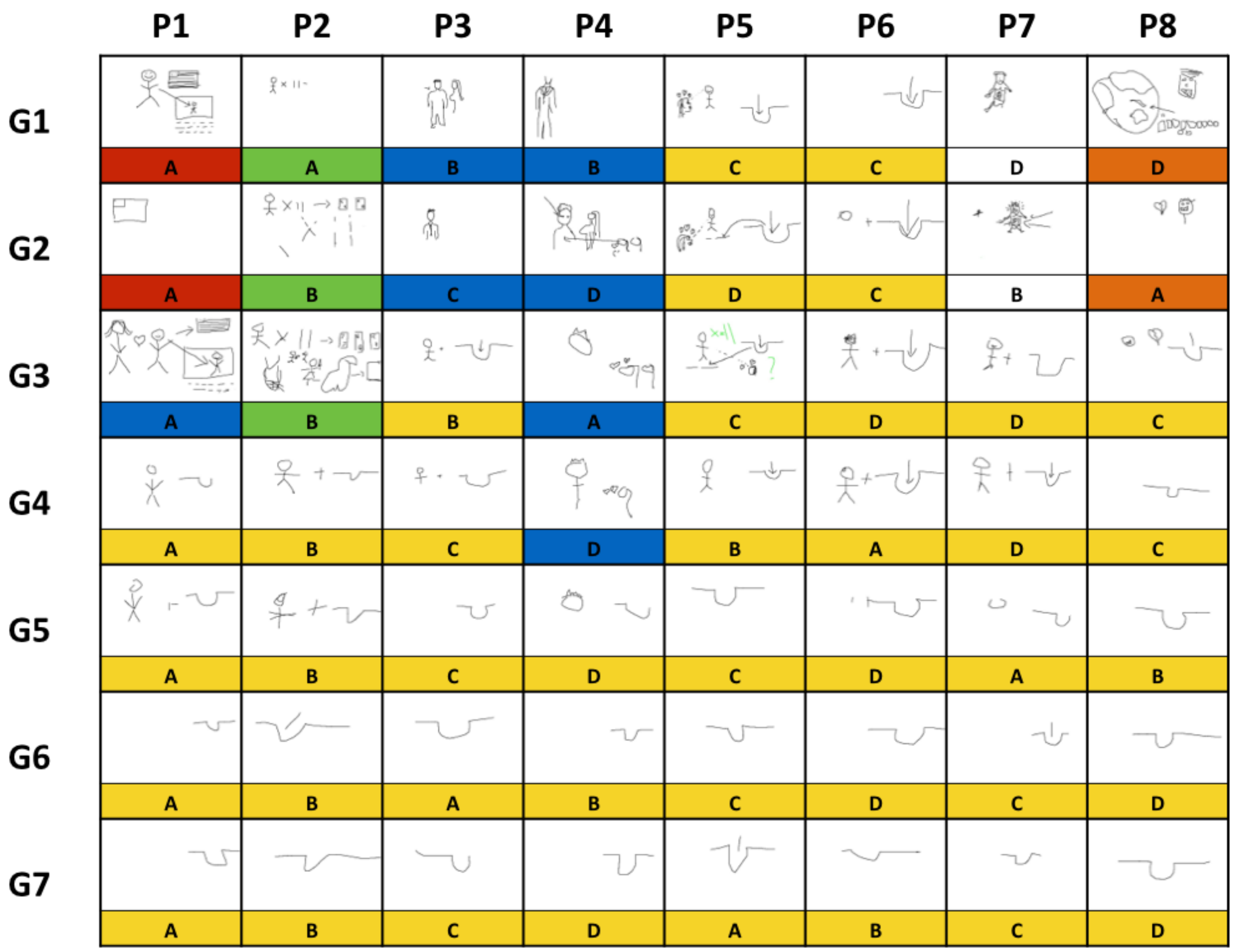
P1 P2 P3 P4 P5 P6 P7 P8
G1 A A B B C C D D
G2 A B C D D C B A
G3 A B B A C D D C
G4 A B C D B A D C
G5 A B C D C D A B
G6 A B A B C D C D
G7 A B C D A B C D
