# Supplementary material for: Cultural selection drives the evolution of human communication systems
Source: Proc Biol Sci. 2014 Aug 7;281(1788):20140488. doi: 10.1098/rspb.2014.0488 (PMC4083785; doi:10.1098/rspb.2014.0488)
Supplement: Tamariz et al SM4 [file rspb20140488supp4.pdf]

SUPPLEMENTARY MATERIALS 4

Given any set of parameter values, we can evaluate the choice of representation by each player at each generation. In contrast to the Soap Opera example (Figures 1 and 2a) where a strong Content bias overpowers all other influences, the item Microwave (Figure 2b and Supplementary Materials 2) illustrates the effects of Content bias, Coordination bias and Memory. The likelihood of this data-structure is maximized when the model parameters are $m = 6, c = -0.2, \tau = D, b = 0.4, \mu = 0.02$. We focus on the representational choice of Player P1 in Generation G6. Their egocentric and allocentric histories at this point are green, green, yellow variant and red, yellow, blue variant respectively. If they choose something other than the red, green, blue or yellow variant, their choice is ahistorical and so has the fixed probability $\mu\varphi(x) = 0.02/8$. The relative frequencies $f\left(x|h_{|E,m}\right), f\left(x|h_{|A,m}\right)$ of $x = red, green, blue, yellow$ in egocentric and allocentric memory are $(0,\frac{1}{3})$, $(\frac{2}{3},0)$, $(0,\frac{1}{3})$, $(\frac{1}{3},\frac{1}{3})$ respectively. Since the Content-biased variant is historical, occurring both in egocentric and allocentric history, Content bias applies, with $\beta = 0.4, \bar{\beta} = 0.6$. The actual choice $yellow\ varaint$ has probability 0.59, while its three historical competitors $red, green, yellow\ variant$ have probabilities of 0.08, 0.24, 0.08 respectively. This model correctly predicts the Player's choice of variant. These values are illustrated in Figure SM4.

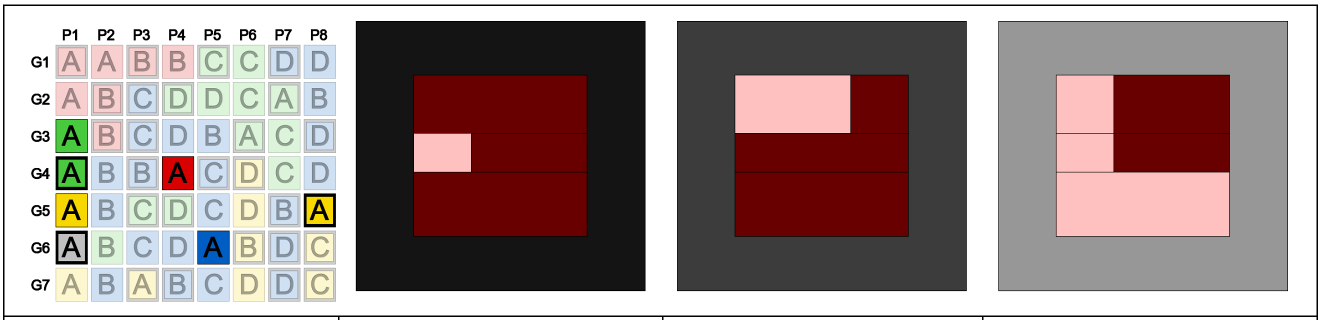

(a)	(b)	(c)	(d)

SM4. Data structure reflecting the changing frequencies of the variants used to communicate *Microwave* given in (a). This data structure illustrates the Variant options available to P1 at G6 (grey fill) with an Egocentric memory of 3, an Allocentric memory of 3, a Coordination bias of -0.2 and a Content bias of 0.4 in favour of the yellow variant. The history h of variants produced or seen by P1 and retained in memory for production are shown in solid color. The probabilities of P1 producing the red, green or yellow variant are given in (b), (c) or (d) respectively. The three horizontal bars express the impact of Egocentric memory (top), Allocentric memory (middle) and Content Bias (bottom). Coordination Bias determines the relative height of the top two bars ($\bar{\beta}\bar{\gamma}$ and $\bar{\beta}\gamma$), balancing Egocentric and Allocentric relative frequencies for a variant. In the present example, we have a ratio of 3:2 in favour of reusing variants from Egocentric memory. The Content Bias determines the relative proportions of the third bar β and the first two combined ($\bar{\beta}\bar{\gamma} + \bar{\beta}\gamma = \bar{\beta}$). If there is no Content bias, or the biased variant has not been encountered, the bottom bar is absent. Here, Content Bias is 0.4 and P1 has encountered the biased yellow variant, so the bottom bar has height 0.4 while the upper two bars fill the remaining 0.6. In an unbiased Drift model, there is no bottom bar (as there is no Content Bias), and the Egocentric and Allocentric bars have equal height. The width of light colour in the top two bars shows the relative frequency of the variant in the corresponding memory: red variant occupies 1/3 of Allocentric memory (b) having been seen once, and green variant occupies 2/3 of Egocentric memory (c) having been produced twice. The bottom bar is all light colour if the variant is Content-biased ($\delta_{\tau}^{x} = 1$) and all dark otherwise ($\delta_{\tau}^{x} = 0$). In (d), we see that yellow variant was produced once (1/3 of Egocentric memory), seen once (1/3 of Allocentric memory), and is the biased variant. The area of light colour is the contribution of everything except mutation to the final probability. The perimeter reflects in shades of grey (black=0.0, white=1.0) the total probability $P(x|h)$ of

selecting the variant as calculated using Equation (1): for red, green and yellow variants these are 0.08, 0.24 and 0.59.
