# Supplementary material for: Cultural selection drives the evolution of human communication systems
Source: Proc Biol Sci. 2014 Aug 7;281(1788):20140488. doi: 10.1098/rspb.2014.0488 (PMC4083785; doi:10.1098/rspb.2014.0488)
Supplement: Tamariz et al SM5 [file rspb20140488supp5.pdf]

SUPPLEMENTARY MATERIALS 5

We describe the data-structure for the item *Microwave* (Figure 2b and Supplementary Materials 2), the breakdown of the likelihood calculation for the optimal parameter settings, and the likelihood contour over the 121 possible bias levels examined. This sample data structure supports a role for biased selection: both coordination and content bias play a role, but the selective pressure exerted by content bias is stronger. Below (SM5) we show the data structure for *Microwave*, the breakdown of probabilities under the best fit model, and how the likelihood is affected by variation in the two bias levels (for a memory size of 6).

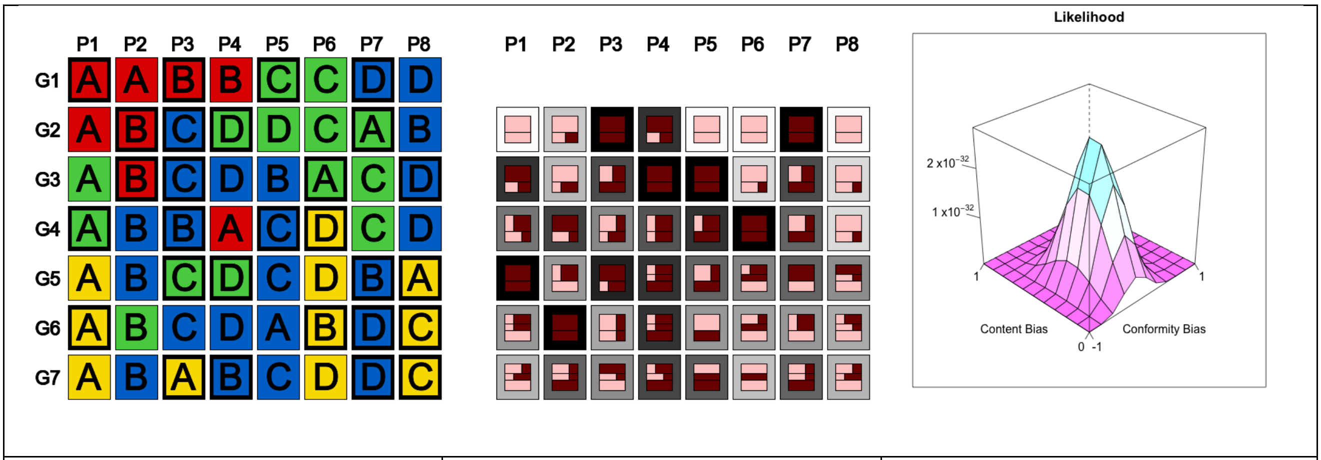

(a)	(b)	(c)

SM5. Panel (a) is the data structure for *Microwave* within one microsociety. Panel (b) shows the components to the calculation corresponding to each data point in Generations G2-7, in the form detailed in Figure 3, for the parameter settings returning the greatest likelihood of data. Panel (c) shows how the likelihood of the data varies with Content and Coordination bias, for a fixed Memory size of 6. The peak likelihood indicates that a biased model best fits the data in this data structure, with Coordination Bias of -0.2 and Content Bias of 0.4.
